# NADES-Extracted SunGold Kiwifruit Polyphenols as Functional Ingredients in Antioxidant-Enriched Yoghurt

**DOI:** 10.3390/antiox15091126

**Published:** 2026-09-05

**Authors:** Rifqah Azzahra Naulidia, Michelle Ji Yeon Yoo

**Affiliations:** School of Science, Faculty of Health and Environmental Sciences, Auckland University of Technology, Private Bag 92006, Auckland 1142, New Zealand; mmt1991@autuni.ac.nz

**Keywords:** SunGold kiwifruit, natural deep eutectic solvent, polyphenols, yoghurt, antioxidant activity, physicochemical properties, functional food

## Abstract

Natural deep eutectic solvents (NADES) offer a potentially food-compatible alternative for extracting plant polyphenols, although their direct application in yoghurt remains limited. This study evaluated the antioxidant and physicochemical properties of yoghurt fortified with SunGold kiwifruit extracts obtained using a selected choline chloride and glycerol NADES, with water extraction used for comparison. The phenolic profile of the NADES extract was characterised using LC-MS/MS. Both extracts were incorporated before (PRE) or after (POS) fermentation at concentrations of 10%, 20%, and 30% (*v*/*v*). The extracts and fortified yoghurts were analysed for total phenolic content (TPC), total flavonoid content (TFC), DPPH radical scavenging activity, FRAP, CUPRAC, and physicochemical properties. LC-MS/MS tentatively annotated several phenolic compounds in the NADES extract, with catechin showing the largest peak area among the annotated compounds. The water extract showed higher TPC and DPPH activity, whereas the NADES extract showed higher TFC, FRAP, and CUPRAC values. Increasing extract concentration generally increased the measured bioactive properties of the yoghurts, although the magnitude of change varied among assays and treatments. Water extract-fortified yoghurts generally showed higher TPC and DPPH values, while NADES extract-fortified yoghurts showed higher TFC, FRAP, and CUPRAC values. Extract type, fortification stage, and concentration also influenced pH, °Brix, viscosity, colour, and syneresis. Higher fortification concentrations reduced viscosity and increased syneresis, while low-concentration NADES extract-fortified yoghurts retained physicochemical properties closer to those of the control. Overall, the NADES extract showed potential as an ingredient for yoghurt fortification. Further studies are required to evaluate sensory acceptability, storage stability, starter culture viability, and gastrointestinal bioaccessibility.

## 1. Introduction

The incorporation of plant-derived polyphenols into food products has long been investigated as an approach to developing functional foods. Dairy products are commonly used for this purpose because they are widely consumed, nutritionally valuable, and generally well accepted by consumers [1]. Among dairy products, yoghurt is particularly suitable as a carrier for plant-derived bioactive compounds because of its protein-rich matrix, relatively simple production process, and formulation flexibility [2]. Fortification with polyphenol-rich extracts may enhance the functional properties of yoghurt, particularly its antioxidant capacity.

SunGold kiwifruit (*Actinidia chinensis* var. *chinensis*) is known for its yellow flesh, sweet flavour, and high nutritional value [3]. Its total phenolic and flavonoid contents have been reported as 113.09 ± 3.22 mg GAE/100 g and 16.33 ± 3.06 mg CTE/100 g, respectively, exceeding those reported for the Hayward cultivar, commonly known as green kiwifruit [4]. Identified polyphenols include quercetin (10.16 mg/44.48 g), catechin (7.68 mg/44.8 g), chlorogenic acid (6.92 mg/44.8 g), epicatechin (5.74 mg/44.8 g), and epigallocatechin (3.22 mg/44.8 g), which may contribute to its antioxidant properties [5]. Previous studies have also reported DPPH and FRAP antioxidant activities of 6.99 ± 0.17 µmol/g and 11.71 ± 0.10 µmol/g, respectively [4].

The recovery of these compounds commonly involves organic solvents, such as methanol and ethanol. Although effective, these solvents may raise environmental and safety concerns and generally require removal before the extracts can be incorporated into food products, limiting their suitability for clean-label formulations [6]. Natural deep eutectic solvents (NADES) have emerged as promising green alternatives because of their low volatility, adjustable polarity, and ability to solubilise and stabilise phenolic compounds. NADES formulated from food-compatible components may also allow direct incorporation of the extracts into food products without complete solvent removal [7].

To date, limited information is available on the incorporation of NADES-extracted SunGold kiwifruit polyphenols into yoghurt. Most available research has focused on extraction performance, leaving uncertainty about whether NADES can retain or improve the functionality of extracted polyphenols after incorporation into a complex food matrix. NADES components may influence antioxidant activity and polyphenol stability while also affecting yoghurt fermentation, gel structure, and physicochemical quality. Their effects may vary according to extract type, fortification concentration, and whether fortification occurs before or after fermentation [8]. Therefore, this study evaluated yoghurts fortified with NADES-extracted SunGold kiwifruit polyphenols, using water extract for comparison. The phenolic profile of the NADES extract was characterised using LC–MS/MS to support interpretation of its antioxidant properties. The effects of extract type, fortification stage, and concentration on the measured antioxidant and physicochemical properties of the fortified yoghurts were also evaluated.

## 2. Materials and Methods

### 2.1. Materials

The chemicals and reagents used in this study were purchased from Sigma-Aldrich (St. Louis, MO, USA), including choline chloride, glycerol, gallic acid, catechin, Trolox, Folin–Ciocalteu reagent, sodium carbonate (Na_2_CO_3_), sodium nitrite (NaNO_2_), aluminium chloride (AlCl_3_), sodium hydroxide (NaOH), 2,2-diphenyl-1-picrylhydrazyl (DPPH), methanol, ferric chloride (FeCl_3_), 2,4,6-tri(2-pyridyl)-s-triazine (TPTZ), hydrochloric acid (HCl), copper(II) chloride (CuCl_2_), ammonium acetate, neocuproine, ethanol, and formic acid. Distilled water and Milli-Q water were used for reagent preparation and sample dilution. Skim milk powder and commercial yoghurt containing starter cultures were used as the main ingredients for yoghurt preparation.

### 2.2. Extraction of SunGold Kiwifruit Extract Using NADES

Choline chloride:glycerol was selected as a practical NADES system based on the availability of its components, ease of preparation, and practical considerations related to its intended application in yoghurt. This NADES system has also previously been used for the extraction of plant-derived phenolic compounds [9]. Preliminary extraction work was conducted separately to establish consistent working conditions for preparing the extract used in the yoghurt fortification experiments. Based on this preliminary work, the extraction conditions selected for the present study were a choline chloride-to-glycerol molar ratio of 1:1, 50% water content (*w*/*w*), a solid-to-solvent ratio of 1:20 (*w*/*v*), and an extraction time of 20 min. These conditions are reported as selected working conditions and are not presented as a formally validated optimum. The NADES was prepared by mixing choline chloride and glycerol at a molar ratio of 1:1 and heating the mixtures at 100 °C until clear, homogeneous liquids were obtained, following the preparation approach described in a previous study [9]. Water was subsequently added to obtain a final water content of 50% (*w*/*w*).

Freeze-dried SunGold kiwifruit powder was mixed with the prepared NADES at a solid-to-solvent ratio of 1:20 (*w*/*v*). Extraction was conducted in an ultrasonic bath for 20 min at 30–40 °C. Water extraction was performed under the same extraction conditions for comparison. The resulting liquid extracts were subsequently used for yoghurt fortification.

### 2.3. Preparation of SunGold Kiwifruit Extract-Fortified Yoghurts

Yoghurt was produced using a kitchen yoghurt maker (Davis and Waddel, Stevens, New Zealand). The control was prepared by reconstituting 200 g of skim milk powder (Woolworths, Auckland, New Zealand) with 800 g of boiling water (100 °C). The reconstituted milk was cooled in a water bath to 46 °C. Once the target temperature was reached, 2% (*w*/*w*) commercial yoghurt containing starter cultures (De Winkel, Fonterra Brands (NZ) Ltd., Auckland, New Zealand) was added, and the mixture was incubated at 43 °C in a yoghurt maker for 9 h until the pH dropped below 5.0. The yoghurt was stored at 4 °C and analysed within 48 h. Prior to analysis, the yoghurt sample was homogenised using a laboratory mixer (Silverson, Waterside, UK) at 4000 rpm for 2 min [8].

An informal preliminary formulation trial was conducted using extract concentrations of 1%, 5%, 10%, 20%, 30%, and 40% (*v*/*v*) to determine a suitable fortification range. The formulations containing 1% and 5% extract showed minimal perceptible differences in flavour compared with the control, whereas the 40% formulation produced an excessively diluted consistency. Therefore, concentrations of 10%, 20%, and 30% (*v*/*v*) were selected for further evaluation, as they provided perceptible extract incorporation while retaining a yoghurt-like consistency. This preliminary assessment was conducted solely for formulation selection and did not constitute a formal sensory evaluation.

Fortified yoghurts were prepared following the same procedure as the control yoghurt, with extracts added at 10%, 20%, and 30% (*v*/*v*) either before fermentation (PRE) or after fermentation (POS). For PRE treatments, the extracts were added immediately after inoculation of commercial yoghurt to the milk, prior to incubation, while for POS treatments, the extracts were added into the yoghurt during the final homogenisation step after fermentation [8].

Samples were labelled according to extract type, fortification stage, and extract concentration. NADES-PRE and NADES-POS refer to yoghurts fortified with the NADES-prepared SunGold kiwifruit extract before and after fermentation, while Water-PRE and Water-POS refer to yoghurts fortified with the water-prepared SunGold kiwifruit extract before and after fermentation, respectively. The numbers 10, 20, and 30 indicate the extract concentration added to the yoghurt (% *v*/*v*).

### 2.4. Physicochemical Properties Analysis of Fortified Yoghurt

Physicochemical properties of yoghurt, including pH, °Brix number, viscosity, colour, and syneresis were evaluated in this study. Viscosity was measured using rotational rheometer (RSX-SST, Rheo 3000, Brookfield AMETEK, Middleboro, MA, USA) equipped with a concentric cylinder geometry consisting of a cup CCT-25 and an MBT-25F measuring bob over a shear rate range of 0–400 s^−1^. Colour parameters (L*, a*, and b*) were determined using a digital colourimeter (Nix Pro Colour Sensor™, Nix Sensor Ltd., Burlington, ON, Canada). Syneresis was assessed following the method described by Diep et al. [8] with slight modifications. Briefly, 10 g of each yoghurt sample was centrifuged at 1500 rpm for 10 min at 4 °C. The supernatant obtained was weighed and recorded as the expelled whey. Syneresis was calculated using the following equation:

$$\mathrm{Syneresis}\;(\%)\;=\;\frac{Wf}{Wi}\times 100$$

where *Wf* is the final weight of the separated whey (supernatant) and *Wi* is the initial weight of yoghurt sample.

### 2.5. Determination of Bioactive Properties of Raw Extracts and Fortified Yoghurt

The bioactive properties of raw extracts and fortified yoghurt samples were evaluated in terms of total phenolic content (TPC), total flavonoid content (TFC), DPPH radical scavenging activity, FRAP, and CUPRAC assays. Prior to analysis, yoghurt samples were freeze-dried for 48 h and extracted using acidified methanol (methanol containing concentrated HCl) at a ratio of 1:3 (*w*/*v*). The mixture was homogenised at 12,000 rpm for 1 min and stored at −20 °C overnight. Subsequently, the samples were centrifuged at 10,000 rpm for 10 min at 4 °C and filtered through a 0.22 µm syringe filter. The resulting filtrate was collected and used for further analysis [10,11].

Plain NADES without kiwifruit extract was analysed under the same assay conditions to evaluate potential interference from the solvent components. Its absorbance was used for background correction and subtracted from the corresponding absorbance of the NADES extract samples before calculation of TPC, TFC, DPPH, FRAP, and CUPRAC values.

#### 2.5.1. Total Phenolic Content (TPC)

Total phenolic content was quantified using gallic acid as the reference standard. A calibration curve was prepared from gallic acid solutions at concentrations of 0, 1, 2.5, 5, 10, 20, and 40 µg/mL. Briefly, 250 µL of extract or yoghurt sample was transferred into a clean tube and diluted with 3750 µL of distilled water. Then, 250 µL of Folin–Ciocalteu reagent was added, and the mixture was vortexed thoroughly. After 8 min of incubation in the dark, 750 µL of 20% (*w*/*v*) Na_2_CO_3_ was added. The reaction mixture was further incubated in the dark for 2 h. Absorbance was measured at 765 nm using a UV-Vis spectrophotometer (GENESYS™ 150 Vis/UV-Vis Spectrophotometer, Thermo Fisher Scientific, Waltham, MA, USA). The results were expressed as mg gallic acid equivalent per gram sample (mg GAE/g) [12].

#### 2.5.2. Total Flavonoid Content (TFC)

TFC was evaluated using the aluminium chloride colorimetric method according to previous method [12], with minor modifications. Catechin (1000 µg/mL) was used as the reference standard, and calibration curves were prepared over a concentration range of 0–100 µg/mL. For the analysis, 250 µL of each sample was diluted with 3450 µL of distilled water. Then, 150 µL of 5% NaNO_2_ (*w*/*v*) solution was added, and the mixture was vortexed and kept in the dark for 5 min. Following this, 150 µL of 10% AlCl_3_ (*w*/*v*) solution was added and allowed to react for 6 min. Finally, 1 mL of 1 M NaOH was added to terminate the reaction. The absorbance was measured at 510 nm using a spectrophotometer. TFC was expressed as mg catechin equivalent (CE)/g.

#### 2.5.3. DPPH Assay

Before analysis, liquid NADES extract and water extract were freeze-dried for 48 h to obtain dry extracts. The dried extracts were diluted with 50% methanol to obtain various concentrations. Trolox standards were prepared at final concentrations of 1, 2.5, 5, 10, and 20 µg/mL for Trolox. NADES extracts were tested at concentrations of 0.25, 0.5, 1, 2.5 and 5 mg/mL, while water extracts were evaluated at 0.1, 0.25, 0.5, 1, and 2.5 mg/mL. For yoghurt samples, 250 µL of sample was used.

Each sample was mixed with 500 µL of 1 mM DPPH solution and vortexed. The total volume was adjusted to 2.5 mL using 50% methanol. The reaction mixture was incubated in the dark for 30 min, and the absorbance was measured at 517 nm using a UV–Vis spectrophotometer [12]. Methanol was used as the blank to minimise interference from sample colour. %Inhibition was calculated using the following equation:

$$\%\mathrm{Inhibition}\;=\;\frac{\left(Ablank-Asample\right)}{Ablank}\times 100$$

where Ablank is the absorbance of the blank, while Asample is the absorbance of the sample. For raw extracts, DPPH activity was expressed as both IC_50_ and mg Trolox equivalent per gram sample (mg TE/g). The IC_50_ value of each sample was calculated using regression analysis of the inhibition curve plotted against concentration. Meanwhile, for yoghurt samples, the results were expressed as mg TE/g.

#### 2.5.4. FRAP Assay

FRAP antioxidant activity was measured using Trolox as the reference standard at concentrations of 0, 0.5, 1, 2.5, 5, 10, and 25 µg/mL. The FRAP reagent was prepared by mixing 300 mM acetate buffer (pH 3.6), 20 mM FeCl_3_, and 10 mM 2,4,6-tri(2-pyridyl)-s-triazine (TPTZ) dissolved in 40 mM HCl in a ratio of 10:1:1. For the assay, 40 µL of sample was added to a clean tube and mixed with FRAP reagent (1200 µL) [12]. The reaction mixture was incubated in the dark for 20 min. Absorbance was then measured using a UV–Vis spectrophotometer at 593 nm. The results were expressed as mg TE/g.

#### 2.5.5. CUPRAC Assay

The CUPRAC assay was performed following the method of [13]. Briefly, 1 mL of Trolox standard or sample was combined with 1 mL of CuCl_2_ solution (0.01 M), 1 mL of ammonium acetate (1 M, pH 7), 1 mL of neocuporine (0.0075 M) prepared in 96% ethanol and 100 µL of Milli-Q water. The reaction mixture was kept in the dark for 5 min, after which the absorbance was recorded at 450 nm using a UV–Vis spectrophotometer. CUPRAC values were expressed as mg TE/g.

### 2.6. Identification of Polyphenols of the NADES Extract Using LC-MS/MS

LC-MS/MS analysis was conducted to characterise the phenolic profile of the NADES extract using Thermo Fisher Scientific Stellar mass spectrometer coupled to a Thermo Scientific Vanquish UHPLC system (Thermo Fisher Scientific, San Jose, CA, USA). Chromatographic separation was performed using a reversed-phase C18 column with the mobile phase consisting of water containing 0.1% formic acid as solvent A and acetonitrile containing 0.1% formic acid as solvent B. Gradient elution was conducted for 40 min at a flow rate of 0.30 mL/min. The injection volume was 1 µL. The analysis was carried out in untargeted mode to provide an overview of the compounds present in the extract. Putative identification of phenolic compounds was based on mass spectral data, retention behaviour, and comparison with available databases and the literature. The resulting profile was used as supporting compositional information to interpret the functional activity of the NADES extract.

### 2.7. Statistical Analysis

Statistical analysis was conducted using Minitab Statistical Software version 22. Each analysis was conducted using three independently prepared samples, with two technical measurements performed for each sample. Results are presented as mean ± standard deviation based on the three independent samples (*n* = 3). One-way analysis of variance (ANOVA), followed by Tukey’s post hoc test, was used to compare the extracts, control yoghurt, and fortified yoghurt treatments. Correlations between TPC, TFC, and antioxidant activities were examined using Pearson’s correlation coefficient. To further evaluate the effects of the fortification variables, three-way ANOVA was performed only on the fortified yoghurt samples, using extract type, fortification stage, and extract concentration as fixed factors. The model included all main effects, two-way interactions, and the three-way interaction. Model assumptions were assessed separately for each response. Residual normality was evaluated using the Anderson–Darling test and normal probability plots, while homogeneity of variance was assessed using Levene’s test and residual-versus-fitted plots. Residual versus order plots were examined for independence, and standardised residuals were inspected for potential outliers. No substantial violations of the model assumptions were identified. Partial eta squared (partial η^2^) was calculated from the adjusted sums of squares to quantify the magnitude of each main and interaction effect. Statistical significance was set at *p* < 0.05.

## 3. Results and Discussion

### 3.1. Bioactivity of SunGold Kiwifruit Raw Extracts

The bioactive properties of raw SunGold kiwifruit extracts, including TPC, TFC, DPPH, FRAP, and CUPRAC, are shown in Table 1. The water extract showed significantly higher TPC than the NADES extract, with values of 9.338 ± 0.128 and 4.200 ± 0.163 mg GAE/g, respectively. This indicates that, under the extraction conditions used in this study, water recovered more Folin–Ciocalteu-reactive compounds from mature SunGold kiwifruit than the choline chloride:glycerol-based NADES system.

This result contrasts with previous work in which choline chloride:glycerol-based NADES showed high phenolic extraction efficiency from thinned young kiwifruit, with TPC reaching 105.37 ± 1.2 mg GAE/g DW under ultrasound-assisted extraction conditions [14]. The difference may be related to fruit maturity, phenolic profile, and extraction conditions, as the present study used mature SunGold kiwifruit rather than thinned young fruit. Solvent selectivity may also contribute to this difference. Water may extract a broader range of water-soluble phenolic and reducing compounds that respond strongly in the Folin–Ciocalteu assay, whereas NADES extraction depends strongly on solvent polarity, viscosity, hydrogen-bonding interactions, and compatibility with the plant matrix [7]. Similar matrix-dependent behaviour has also been reported in other plant systems, where water extracts showed higher TPC than glucose-citric acid-based NADES extracts [15].

The NADES extract showed significantly higher TFC than the water extract, with values of 1.841 ± 0.090 and 0.996 ± 0.061 mg CE/g, respectively. In general, TFC values were lower than TPC values, which is expected because the aluminium chloride colorimetric assay responds more selectively to flavonoid compounds capable of forming complexes with aluminium ions. This response is mainly associated with specific structural features of flavones and flavonols, including the C-4 keto group, C-3 or C-5 hydroxyl groups, and ortho-dihydroxyl groups in the flavonoid structure [16]. The higher TFC in the NADES extract suggests that the choline chloride:glycerol-based NADES system may have favoured the extraction of flavonoid-type compounds from SunGold kiwifruit. This agrees with previous findings where flavonoid-related compounds, including procyanidins, catechin, epicatechin, and quercetin derivatives, were identified as important phenolic constituents recovered from thinned young kiwifruit using NADES-based extraction [14]. This selectivity may be associated with the hydrogen-bonding network, polarity, and hydrogen bond donor/acceptor interactions of NADES systems [17].

DPPH radical scavenging activity showed a similar trend with TPC. The water extract exhibited significantly higher DPPH activity than the NADES extract, with values of 11.511 ± 0.120 and 9.867 ± 0.025 mg TE/g, respectively. This was consistent with the IC_50_ results in Table 2, where the water–SunGold kiwifruit extract showed a significantly lower IC_50_ value than the NADES–SunGold kiwifruit extract, with values of 0.633 ± 0.018 and 2.780 ± 0.480 mg/mL, respectively. Since lower IC_50_ values indicate stronger radical scavenging activity, the water extract showed stronger DPPH radical scavenging potency than the NADES extract.

Trolox was used as the reference antioxidant standard due to its strong radical scavenging activity, mainly associated with its phenolic hydroxyl group and the electron-donating effect of the adjacent methyl group [18]. Both kiwifruit extracts showed higher IC_50_ values than Trolox, indicating lower radical scavenging potency than the pure reference antioxidant. The IC_50_ value of the water extract was within the range reported for polyphenol-enriched extracts from thinned unripe kiwifruit, where DPPH IC_50_ values ranged from 0.265 to 0.644 mg/mL [19]. The higher IC_50_ value observed for the NADES extract may be related to differences in fruit maturity, cultivar, extraction conditions, and phenolic composition. Kiwifruit total phenolics and DPPH radical scavenging activity have been shown to vary with harvest stage, with earlier developmental stages generally showing stronger radical scavenging activity [20]. In this study, the stronger DPPH activity of the water extract may therefore be associated with its higher TPC value, suggesting that water recovered more DPPH-reactive compounds, including phenolics and other reducing compounds such as ascorbic acid.

FRAP and CUPRAC showed different trends from TPC and DPPH. The NADES extract had significantly higher FRAP than the water extract, with values of 9.039 ± 0.401 and 6.529 ± 0.348 mg TE/g, respectively. Similarly, CUPRAC was much higher in the NADES extract than in the water extract, with values of 95.565 ± 0.215 and 38.764 ± 0.538 mg TE/g, respectively. These results indicate that the NADES extract had stronger reducing capacity, despite having lower TPC and DPPH activity than the water extract.

This difference may be explained by the different antioxidant mechanisms measured by each assay. DPPH mainly reflects radical scavenging activity, whereas FRAP and CUPRAC are based on electron transfer and reducing power [21]. Therefore, compounds that respond strongly in FRAP and CUPRAC may not necessarily show the same response in DPPH. A similar assay-dependent pattern was reported on *Chenopodium album* extracts prepared using different solvents with methanol extract showing the highest DPPH and ABTS radical scavenging activities, whereas the ethanol extract showed the highest CUPRAC value and the water extract showed the highest FRAP value [22]. This indicates that the extract with the strongest radical scavenging activity does not always exhibit the highest reducing power. In the present study, the high FRAP and CUPRAC values of the NADES extract suggest that the choline chloride:glycerol-based NADES system may have selectively extracted compounds with strong electron-donating capacity, particularly flavonoid-type compounds, which is consistent with its higher TFC value.

### 3.2. Polyphenol Profile of the NADES Extract

The phenolic profile of the NADES-based SunGold kiwifruit extract was evaluated using LC–MS/MS in negative ionisation mode, as shown in Table S1. Several phenolic compounds and phenolic derivatives were tentatively annotated, including flavanols, flavonol glycosides, hydroxycinnamic acids, hydroxybenzoic acids, and proanthocyanidins. Among the annotated compounds, catechin showed the largest peak area under the LC-MS/MS conditions used, suggesting that flavanol-type compounds were one of the major phenolic groups present in the NADES-based extract. This may be partly related to the acidic nature of the choline chloride:glycerol-based NADES used in this study, as catechins have been reported to be more stable under acidic conditions, particularly at pH below 4.0 [23]. However, peak areas were used only as relative instrumental responses and not as quantitative measures of compound concentration.

This finding is supported by a previous study on kiwifruit phenolic extraction using a choline chloride:glycerol-based deep eutectic solvent at a molar ratio of 1:2 with 20% water addition. In that study, the phenolic profile was also dominated by flavan-3-ols, particularly procyanidin B2, procyanidin B1, and epicatechin, along with neochlorogenic acid as the major phenolic acid [14]. Similarly, a previous study on polyphenol extraction from SunGold kiwifruit peel and flesh reported the presence of several phenolic compounds, including quercetin, catechin, chlorogenic acid, epicatechin, kaempferol, epigallocatechin, caffeic acid, rutin, isoquercetin, and ferulic acid [5].

Catechin and other related flavanol compounds, such as epicatechin 7-O-glucuronide and (+)-gallocatechin, have been widely reported for their antioxidant properties, mainly through free radical scavenging, metal ion chelation, and modulation of oxidative stress-related pathways. Thus, the antioxidant activities observed in this study may be partly attributed to the presence of these flavanol compounds. However, these bioactivities are unlikely to result from a single compound alone, as the bioactivities of the NADES-based extract are likely associated with the combined effects of multiple phenolic compounds. This is supported by a previous study showing that synergistic interactions among polyphenols can contribute to their biological activities, including antioxidant and other health-related effects [24].

### 3.3. Bioactivity of Yoghurt Fortified with SunGold Kiwifruit Extracts

After incorporation into yoghurt, the fortified samples generally showed higher bioactive properties than the control yoghurt, although their values were considerably lower than those of the corresponding raw extracts. The magnitude of these differences varied among assays and treatments. Several low-concentration treatments showed only modest absolute changes, whereas larger increases were observed at higher concentrations, particularly for FRAP and CUPRAC in NADES extract-fortified yoghurts. As shown in Table 3, the fortified yoghurts showed TPC values ranging from 0.136 to 0.390 mg GAE/g, TFC from 0.030 to 0.117 mg CE/g, DPPH from 0.557 to 1.194 mg TE/g, FRAP from 0.317 to 1.244 mg TE/g, and CUPRAC from 0.718 to 2.815 mg TE/g. The control yoghurt showed TPC, DPPH, FRAP, and CUPRAC values of 0.118 mg GAE/g, 0.542 mg TE/g, 0.260 mg TE/g, and 0.720 mg TE/g, respectively, while TFC was not detected. The measurable bioactivity in the control yoghurt may be related to the intrinsic antioxidant and reducing capacity of yoghurt components, including milk proteins, vitamins, minerals, lactic acid bacteria, and metabolites produced during fermentation [8,25,26].

In general, increasing extract concentration improved the bioactive properties of fortified yoghurts, although the dominant extract type differed depending on the measured response. Water-fortified yoghurts generally showed higher TPC and DPPH activity than NADES extract-fortified yoghurts, following the trend observed in the raw extracts. Water-PRE-30 showed the highest TPC and DPPH values, followed by Water-POS-30. In contrast, NADES extract-fortified yoghurts showed higher TFC, FRAP, and CUPRAC values. The highest TFC was observed in NADES-PRE-30, followed by NADES-POS-30, while the highest FRAP and CUPRAC values were observed in NADES-POS-30, followed by NADES-POS-20 and NADES-PRE-30. This suggests that the bioactive response of fortified yoghurt was influenced by extract type, extract concentration, fortification stage, and the antioxidant mechanism measured.

The lower values observed in fortified yoghurts compared with the raw extracts may be attributed to dilution of the extracts within the yoghurt matrix and reduced accessibility of bioactive compounds after incorporation. Phenolic and flavonoid compounds may interact with yoghurt matrix components, particularly milk proteins such as casein, through hydrogen bonding, hydrophobic interactions, and other non-covalent or covalent interactions. These interactions may entrap bioactive compounds within the yoghurt gel network, reduce their extractability, and limit their availability to react with colorimetric reagents or antioxidant radicals [27,28].

A previous study on tamarillo polyphenols showed that chlorogenic acid, kaempferol-3-rutinoside, delphinidin-3-rutinoside, and pelargonidin-3-rutinoside interacted with bovine serum albumin in fresh milk cream, resulting in fluorescence quenching, changes in binding behaviour, and altered antioxidant capacity [29]. These findings suggest that fruit-derived polyphenols may bind to milk proteins and become less freely detectable or functionally available within dairy matrices. Similarly, in tamarillo polyphenol-loaded cubosome yoghurt, bioactive compounds were suggested to interact with yoghurt molecules such as proteins and fats, which may influence their measured antioxidant activity [28]. Yoghurt fortified with date polyphenols showed that polyphenol–casein interactions may make phenolic extraction and quantification more difficult [30]. A comparable reduction in measurable flavonoids was also reported in yoghurt fortified with green banana peel polyphenol extract, where TFC values in fortified yoghurt were much lower than those of the original plant material despite increasing with fortification concentration [31]. In addition, storage and digestion may further influence the release, stability, and detectability of bound phenolic compounds, as reported in yoghurt fortified with Bengal currant polyphenols [32]. These findings support the idea that the yoghurt matrix can reduce the apparent recovery and activity of phenolic and flavonoid compounds after fortification. Future studies should evaluate the storage stability and digestion behaviour of fortified yoghurts with SunGold kiwifruit extracts to better understand changes in their measurable bioactivity over time.

In terms of extract type, the NADES and water extracts may have contained different polyphenol and flavonoid profiles, which could explain the differences observed in their bioactive responses. Increasing extract concentration generally resulted in higher measured TPC, TFC, and antioxidant assay values, although the magnitude of these increases varied among assays and treatments, with some statistically significant differences being numerically modest. Relative to the control yoghurt, Water-PRE-30 showed approximately 230% higher TPC and 120% higher DPPH activity, while NADES-POS-30 showed approximately 379% higher FRAP and 291% higher CUPRAC values. These results indicate that larger changes occurred in selected high-concentration treatments than in several low-concentration treatments. Since these values were obtained using chemical antioxidant assays, they should not be interpreted as evidence of improved bioaccessibility or health benefits.

The effect of fortification stage also differed depending on the measured response. TPC and TFC were generally higher when the extracts were added before fermentation than after fermentation, which may be associated with differences in the interaction between the bioactive compounds and yoghurt matrix during gel formation. A similar explanation was suggested by Diep et al. [28], who reported that pre-fermentation fortification allowed encapsulated polyphenols to interact with the yoghurt matrix during fermentation, potentially influencing their stability or release. These mechanisms were not directly evaluated in the present study and should therefore be considered possible interpretations rather than established explanations.

The trends in TPC and TFC were not fully consistent with those of the antioxidant assays, indicating that higher measured phenolic or flavonoid contents did not necessarily correspond to higher antioxidant values. Antioxidant responses may depend on the composition and structural characteristics of phenolic compounds, including their hydroxylation and methoxylation patterns, reducing potential, and interactions with the food matrix [33]. POS fortification generally produced higher FRAP and CUPRAC values than PRE fortification. This pattern may be associated with differences in the exposure of antioxidant-active compounds to fermentation conditions and the yoghurt matrix. Since compound stability and polyphenol–matrix interactions were not directly measured, the underlying mechanisms could not be established. A similar pattern was reported for olive leaf-fortified yoghurt, where post-fermentation addition resulted in higher antioxidant activity [25].

### 3.4. Correlation Between TPC, TFC, and Antioxidant Activities of Raw Extracts

Pearson correlation analysis was performed to examine the relationships between TPC, TFC, and antioxidant activities in the raw NADES and water extracts. The correlations should be interpreted cautiously because they were based on only two independent extraction systems with replicate measurements. Therefore, the coefficients may largely reflect the differences between the two extraction systems rather than robust relationships across a broad range of independent samples.

An apparent negative correlation was observed between TPC and TFC (r = −0.983), as shown in Figure S1, indicating that the extract with higher TPC did not necessarily contain higher flavonoid content. Although several previous studies have reported an apparent positive correlation between TPC and TFC, an opposite trend has also been observed. For example, previous study reported an apparent negative correlation between TPC and TFC in *Solanum scabrum* extracts (r = −0.998), suggesting that extracts with higher phenolic content may not necessarily have higher flavonoid content [34]. This may occur because phenolic compounds are not limited to flavonoids, but also include non-flavonoid phenolics, such as benzoic acid, cinnamic acid, stilbenes, tannins, and lignins [35]. Therefore, the negative correlation observed in the present study may reflect differences in the dominant phenolic subclasses extracted by water and NADES.

The relationship between TPC, TFC, and antioxidant activities was assay-dependent, as shown in Figure S2. TPC showed an apparent positive correlation with DPPH activity (r = 0.994), but apparent negative correlations with FRAP (r = −0.963) and CUPRAC (r = −0.999). In contrast, TFC showed an apparent negative correlation with DPPH (r = −0.990), but apparent positive correlations with FRAP (r = 0.945) and CUPRAC (r = 0.987). This may be consistent with the patterns observed in the fortified yoghurt, showing that antioxidant activity was influenced not only by total phenolic or flavonoid levels, but also by the dominant phenolic subclasses and the assay mechanism. Previous studies have also shown that TPC, TFC, and antioxidant correlations may vary depending on the plant matrix and assay used [36]. This variation may be related to the structural characteristics of phenolic compounds, particularly the number and position of hydroxyl groups, which can influence their radical scavenging activity and reducing power [37].

The different correlation patterns may also reflect solvent selectivity. NADES is considered a tunable solvent system and has been reported to extract phenolic compounds differently from conventional solvents, resulting in variations in phenolic composition and antioxidant responses [38]. For example, in choline chloride-based NADES, the hydrogen bond donor, such as glycerol, can influence the polarity, viscosity, and hydrogen-bonding capacity of the solvent. Since glycerol contains multiple hydroxyl groups, it can interact with phenolic compounds through hydrogen bonding, which may affect the solubility, recovery, and antioxidant activity of individual phenolics [39,40]. Therefore, the observed association between TFC and FRAP/CUPRAC may reflect differences in the compounds recovered by the two extraction systems. However, this interpretation remains preliminary because the analysis included only two independent extraction systems.

To further evaluate the differences among antioxidant responses, Pearson correlation analysis was conducted between antioxidant assays, as shown in Figure S3. FRAP and CUPRAC showed an apparent positive correlation (r = 0.966), which may be explained by their similar electron-transfer-based reducing mechanisms. FRAP is based on the reduction of Fe(III) to Fe(II) (Benzie and Strain), whereas CUPRAC is based on the reduction of Cu(II)-neocuproine to Cu(I)-neocuproine [41]. In contrast, DPPH showed apparent negative correlations with FRAP (r = −0.965) and CUPRAC (r = −0.996), suggesting that radical scavenging activity did not follow the same pattern as reducing capacity in the raw SunGold kiwifruit extracts.

These findings are consistent with previous studies showing that antioxidant assays reflect different reaction mechanisms. Ref. [42] reported that DPPH and ABTS were clustered as radical scavenging assays, whereas TRP, FRAP, and CUPRAC were grouped as reducing power assays. Similarly, Ref. [43] reported that DPPH may represent antioxidant attributes different from those measured by reducing power-based assays. This difference may be attributed to the reaction characteristics of DPPH, which involves scavenging of a stable free radical through hydrogen atom transfer or electron transfer, and can be influenced by reaction kinetics, solvent compatibility, and steric accessibility to the radical site. Thus, antioxidants that are effective in reducing Fe(III) or Cu(II) may not necessarily show proportional activity in the DPPH assay [37,44].

Although some correlation coefficients were high, the limited number of independent extraction systems restricts the reliability and broader interpretation of these relationships. In addition, the relatively low coefficients of determination observed in some regression plots indicate limited explanatory strength. Therefore, these findings should be regarded as preliminary associations within the present dataset rather than definitive relationships between phenolic content and antioxidant activity.

### 3.5. Physicochemical Properties of Fortified Yoghurts

#### 3.5.1. pH

The pH values of the control and fortified yoghurts are presented in Table 4. The control yoghurt had a pH of 4.583, while the fortified yoghurts ranged from 4.268 to 4.687. Yoghurt is produced through the fermentation of milk by lactic acid bacteria, mainly through the conversion of lactose into lactic acid, which typically results in a final pH of around 4.6. The final acidity is an important quality parameter because it influences the sourness, flavour acceptability, and overall characteristics of yoghurt. Less acidic yoghurt may be preferred by some consumers due to its milder sour taste [45].

NADES extract-fortified yoghurts generally showed higher final pH values than water extract-fortified yoghurts. This difference may be partly associated with the initial pH of the extracts. The NADES extract had a pH of 4.00 ± 0.017, while the water extract had a slightly lower pH of 3.93 ± 0.021. The lower pH of the water extract may therefore have contributed to the lower final pH of the corresponding yoghurts. Previous studies have reported pH values between 3.0 and 4.0 for kiwifruit peel, flesh, and seed powders from different cultivars [46]. The acidic nature of SunGold kiwifruit extract may also be associated with its naturally occurring organic acids. Citric acid has been identified as its predominant organic acid, followed by ascorbic acid, with malic, tartaric, and oxalic acids also reported [47].

The effects of concentration and fortification stage varied according to extract type. In NADES-PRE yoghurts, the final pH increased from 4.625 at 10% fortification to 4.687 at 30% fortification. This pattern may indicate differences in acidification when the NADES extract was present during fermentation. One possible explanation is the contribution of choline chloride and the high solute concentration of the NADES system, which may increase osmotic pressure and influence the activity of lactic acid bacteria. A previous study reported that increased salt concentration reduced the viable counts of *Lactobacillus acidophilus* through osmotic stress and caused structural changes in bacterial proteins under acidic conditions [48]. This mechanism may partly explain the higher final pH of NADES-PRE yoghurts than NADES POS yoghurts. In NADES POS samples, fermentation had already occurred before extract addition. The reduction in final pH with increasing NADES concentration may therefore be associated with the intrinsic acidity of the extract rather than changes in acidification. However, real-time pH changes and starter culture viability were not measured in the present study. Therefore, the observed pH differences cannot be directly attributed to osmotic stress or changes in bacterial activity.

Increasing the concentration of the water extract reduced the final pH in both PRE and POS yoghurts, which may be associated with the greater addition of acidic compounds from the kiwifruit extract. In terms of fortification stage, Water-POS yoghurts showed higher final pH values than the corresponding Water-PRE yoghurts at the same concentrations. Previous research on fruit-fortified yoghurt suggests that adding extracts before fermentation may influence acid development because extract-derived soluble compounds are present throughout fermentation [8]. Nevertheless, acidification kinetics and starter culture viability were not measured, so the mechanism underlying the observed differences could not be established.

#### 3.5.2. Brix

Brix measurement was conducted to determine the total soluble solids (TSS) of the yoghurt samples, which mainly reflect sugars but may also include other dissolved compounds such as organic acids, minerals, and extract-derived soluble components [49]. As shown in Table 4, the control yoghurt had a °Brix value of 10.798, while fortified yoghurts ranged from 12.338 to 28.665 °Brix. All fortified samples showed higher °Brix values than the control, indicating that extract addition increased the soluble solid content of the yoghurt matrix.

NADES extract-fortified yoghurts generally showed higher °Brix values than water extract-fortified yoghurts. This may be attributed to the intrinsic °Brix of the extracts, as the NADES extract had a much higher °Brix value than the water extract, at 44.99 ± 0.010 and 4.04 ± 0.043, respectively. Furthermore, NADES solvent components, such as choline chloride and glycerol, may also contribute to the high °Brix values. Previous research reported that neat NADES had refractive index values ranging from 1.4498 to 1.5337 at 298.15 K, while water addition decreased the refractive index of NADES mixtures [50]. Since °Brix is based on refractometric measurement, the higher °Brix values may reflect dissolved non-sugar components from the choline chloride system, together with extract-derived solutes. This may further explain why increasing NADES extract concentration increased the °Brix values, as higher fortification levels contributed more dissolved solids from the NADES solvent system to the yoghurt matrix. Therefore, the higher °Brix values of NADES extract-fortified yoghurts should not be interpreted as an improvement in product quality or as evidence of higher sugar content. The measurements may also reflect the refractive contribution of choline chloride, glycerol, and other non-sugar dissolved components.

In contrast, water extract-fortified yoghurts showed a decreasing °Brix trend as extract concentration increased. This may be associated with the dilution effect of liquid extract fortification, as the water extract had much lower soluble solid content than the yoghurt matrix. A similar effect was reported in noni juice-fortified yoghurt, where increasing juice concentration reduced TSS because the juice contained fewer soluble solids than the yoghurt matrix [51].

In terms of the fortification stage, both NADES and water extract-fortified yoghurts showed higher °Brix values when extracts were added after fermentation. This may be because soluble compounds in PRE samples were present during fermentation and could be partly converted into acids, reducing the final TSS. In contrast, when extracts were added after fermentation, these soluble compounds were more likely to remain in the final yoghurt matrix and contribute to higher °Brix values, as previously explained in tamarillo-fortified yoghurt [8].

#### 3.5.3. Viscosity

Viscosity is an important parameter in yoghurt because it reflects the resistance of the yoghurt matrix to flow and is closely related to texture and consumer acceptability [52]. In this study, viscosity was measured at a shear rate of 350 s^−1^. As shown in Table 4, the control yoghurt had the highest viscosity value of 0.235 Pa·s, while fortified yoghurts ranged from 0.067 to 0.222 Pa·s. Extract fortification generally reduced yoghurt viscosity relative to the control. However, NADES-PRE-10 retained a viscosity of 0.222 Pa·s compared with 0.235 Pa·s for the control and was not significantly different from it, indicating that fortification at this level had a limited effect on viscosity.

Water extract-fortified yoghurts showed lower viscosity than NADES extract-fortified yoghurts at all concentrations, which may be related to the intrinsic properties of the extraction solvents. Choline chloride:glycerol NADES has been reported to possess relatively higher shear viscosity than pure water, while water addition decreased NADES viscosity [53]. Therefore, the lower viscosity of water extract-fortified yoghurts may be associated with the dilution effect of the water extract, which could weaken the yoghurt gel matrix. Furthermore, increasing extract concentration generally decreased viscosity in all fortified yoghurts. This suggests that higher extract addition reduced the resistance to flow, potentially because of the dilution of milk solids and associated changes in protein interactions. A similar pattern was reported in yoghurt fortified with aqueous herb extracts, where viscosity was lower than that of the control across all tested extract concentrations [54]. Since the extracts in the present study were added on a volume-per-volume basis, higher extract concentrations reduced the relative proportion of milk-derived structure-forming components, including casein, fat, and total solids. A lower proportion of these components may result in a weaker protein gel network, reduced interparticle interactions, and consequently lower apparent viscosity in the produced yoghurt [55]. Lastly, the fortification stage also influenced viscosity, as POS samples generally showed lower viscosity than PRE samples for both NADES and water extract-fortified yoghurts. This suggests that adding extracts before fermentation had a stronger effect on gel development, as the extract was present during acidification and casein network formation, as reported by previous research in the tamarillo fortified yoghurt [28].

#### 3.5.4. Colour

In this study, colour was evaluated using L*, a*, and b* values, representing lightness, redness-greenness, and yellowness-blueness, respectively. Compared with the control, fortified yoghurts generally showed lower L* values and less negative a* values, indicating reduced lightness and greenness. The b* values were more dependent on extract type, with water extract-fortified yoghurts showing greater yellowness than the control, while NADES extract-fortified yoghurts showed smaller changes. This is consistent with previous research on yoghurt fortified with *Solanum melongena* phenolic extract, where extract addition significantly affected yoghurt colour parameters [56].

The type of extract influenced the colour response of fortified yoghurts. Water extract-fortified yoghurts generally showed higher b* values, indicating stronger yellowness, whereas NADES extract-fortified yoghurts showed a more pronounced decrease in L*, particularly in PRE samples. This may be related to differences in the extracted colour-contributing compounds, as solvent choice can strongly influence the extraction of plant metabolites due to the chemical properties of both the solvent and plant material [57]. Moreover, different pigment classes can contribute to different colour attributes [58], suggesting that the colour response of fortified yoghurts may depend on the types of pigment or polyphenol subclasses extracted by each solvent.

Increasing extract concentration generally intensified the colour changes, as L* values significantly (*p* < 0.05) decreased in both extracts, indicating darker yoghurt, while a* values generally became less negative, suggesting reduced greenness. The b* response was more treatment-dependent, with water extract-fortified yoghurts showing a clearer increase in yellowness than NADES extract-fortified yoghurts. Furthermore, fortification stage also influenced colour, as PRE samples generally showed lower L* values than POS samples, particularly at higher concentrations, while changes in a* and b* were less consistent. This is consistent with Diep et al. [8], who reported that fortification concentration and timing significantly affected yoghurt colour due to interactions between fruit-derived compounds and the yoghurt matrix during fermentation.

#### 3.5.5. Syneresis

Syneresis was measured to evaluate whey separation and the ability of the yoghurt gel matrix to retain water. A higher syneresis value indicates weaker gel stability and lower water-holding capacity [59]. Syneresis was significantly affected by extract addition, extract concentration, and fortification stage. The control yoghurt showed the lowest syneresis value (5.490%), while fortified yoghurts showed significantly higher values, ranging from 6.604 to 20.835%. The magnitude of this increase was substantial at higher fortification concentrations. For example, Water-POS-30 showed approximately 280% higher syneresis than the control. Unlike statistical significance alone, this large numerical increase indicates a potentially meaningful reduction in the physical stability and water-holding capacity of the yoghurt.

Water extract-fortified yoghurts generally showed higher syneresis than NADES extract-fortified yoghurts. Syneresis increased significantly (*p* < 0.05) with extract concentration, indicating reduced water retention at higher fortification levels. Similar results were reported in yoghurt fortified with strawberry and beetroot liquid extracts, where higher extract levels increased syneresis. This may be due to the higher water content of the water extract, which introduced more free water into the yoghurt matrix and weakened its water-holding capacity [60].

POS samples also tended to show higher syneresis than PRE samples, suggesting that extract addition after fermentation may disrupt the yoghurt gel network formed during fermentation. After fermentation, casein has already formed a three-dimensional gel network. Therefore, post-fermentation addition and mixing of liquid extract may cause structural rearrangement or breakdown of the weak gel matrix, reducing its ability to retain whey. Previous research explained that whey separation is associated with an unstable gel network, which may result from gel matrix rearrangement or damage caused by physical stress such as vibration, cutting, or shearing [61]. This is also consistent with Diep et al. [8], who reported higher syneresis in POS tamarillo-fortified yoghurt than in PRE samples.

### 3.6. Interaction Effects of Extraction Type, Fortification Stage and Concentration on Fortified Yoghurt Properties

A three-way ANOVA was conducted to examine the effects of extract type, fortification stage, and concentration on the bioactive and physicochemical properties of fortified yoghurts. As shown in Table 5, all three factors significantly affected every measured response (*p* < 0.05). However, the significance of their interactions varied among responses, demonstrating that the combined effects of formulation and processing conditions were property dependent. While the ANOVA results identified statistically significant effects, the treatment means in Table 3 and Table 4 show the magnitude of the observed differences.

For the bioactive properties, the extract type × concentration and extract type × fortification stage interactions were significant for all responses (*p* < 0.05), showing that the effects of concentration and fortification stage depended on the extract used. Significant three-way interactions were observed for TFC, FRAP, and CUPRAC, whereas the corresponding interactions for TPC and DPPH were not significant. For the physicochemical properties, significant three-way interactions were observed for pH, viscosity, and all colour parameters, while those for °Brix and syneresis were not significant. Thus, for responses with significant three-way interactions, the concentration-dependent differences between PRE and POS fortification were not consistent across the two extract types.

The partial η^2^ values further demonstrated considerable variation in the magnitude of these effects. Extract type and concentration generally showed the largest effects on TPC, TFC, DPPH, FRAP, and CUPRAC, indicating that the type and amount of extract contributed substantially to variation in the measured bioactive properties. The treatment means generally showed higher bioactive values at increasing extract concentrations, although the magnitude of these changes varied among assays and treatments. Concentration also had particularly large effects on viscosity, colour, and syneresis, demonstrating substantial differences among the 10%, 20%, and 30% formulations. Fortification stage remained influential, although its effect was not consistently as large as those of extract type and concentration.

The magnitude of the three-way interaction was particularly large for viscosity (partial η^2^ = 0.796), substantial for pH (partial η^2^ = 0.398) and the colour parameters (partial η^2^ = 0.217–0.325), but comparatively small for TPC (partial η^2^ = 0.003), °Brix (partial η^2^ = 0.073), and syneresis (partial η^2^ = 0.006). Accordingly, responses with significant and substantial three-way interactions were interpreted using the treatment-level means and Tukey comparisons presented in Table 3 and Table 4 rather than through the main effects independently.

## 4. Conclusions

The selected choline chloride NADES showed potential for extracting SunGold kiwifruit polyphenols and producing an extract suitable for incorporation into yoghurt. LC–MS/MS analysis tentatively annotated several phenolic compounds in the NADES extract, with catechin showing the largest peak area among the annotated compounds. Compared with the water extract, the NADES extract showed higher TFC, FRAP, and CUPRAC values, whereas the water extract showed higher TPC and DPPH activity. Thus, the relative performance of the extracts varied according to the measured bioactive response and antioxidant assay.

Increasing extract concentration generally increased the measured bioactive values, although some statistically significant changes were modest in absolute magnitude. In contrast, reductions in viscosity and increases in syneresis at higher concentrations were comparatively large and may have greater practical relevance to yoghurt texture and physical stability. PRE fortification generally produced higher TPC and TFC values and, in several treatments, higher viscosity and lower syneresis, whereas POS fortification generally produced higher FRAP and CUPRAC values. These findings indicate that the measured properties were influenced by the combined effects of extract type, fortification stage, and concentration, with the magnitude of these effects varying among responses.

The findings are limited to freshly prepared yoghurt analysed within 48 h and antioxidant responses measured before gastrointestinal digestion. Sensory acceptability, refrigerated storage stability, starter culture viability, and gastrointestinal bioaccessibility were not evaluated. Plain NADES was included as a background control for the spectrophotometric assays. However, yoghurt fortified with plain NADES at the corresponding concentrations was not included as a separate formulation control. Therefore, the independent effects of the NADES components on the physicochemical properties of yoghurt could not be determined. A comprehensive comparison of different NADES systems was also not conducted. Consequently, the present findings do not establish choline chloride as the optimal or best-performing NADES for SunGold kiwifruit extraction. Future studies should compare different NADES formulations and evaluate their safety, regulatory suitability, sensory effects, acidification kinetics, starter culture viability, polyphenol stability during storage, gastrointestinal bioaccessibility, and process scale-up.

## Figures and Tables

**Table 1:** TPC, TFC, and antioxidant activities of raw extracts.

Sample	TPC (mg GAE/g)	TFC (mg CE/g)	DPPH (mg TE/g)	FRAP (mg TE/g)	CUPRAC (mg TE/g)
NADES extract	4.200 ± 0.163 ^b^	1.841 ± 0.090 ^a^	9.867 ± 0.025 ^b^	9.039 ± 0.401 ^a^	95.565 ± 0.215 ^a^
Water extract	9.338 ± 0.128 ^a^	0.996 ± 0.061 ^b^	11.511 ± 0.120 ^a^	6.529 ± 0.348 ^b^	38.764 ± 0.538 ^b^

Notes: Values are expressed as mean ± standard deviation (*n* = 3). Different superscript letters within the same column indicate significant differences (*p* < 0.05).

**Table 2:** DPPH IC_50_ values of NADES- and water-extracted SunGold kiwifruit extracts.

Sample	DPPH IC_50_ Value
Trolox (standard)	10.767 ± 0.329 ^c^ (μg/mL)
NADES–SunGold kiwifruit extract	2.780 ± 0.48 ^a^ (mg/mL)
Water–SunGold kiwifruit extract	0.633 ± 0.018 ^b^ (mg/mL)

Notes: Values are expressed as mean ± standard deviation (*n* = 3). Different superscript letters indicate significant differences within the same column (*p* < 0.05). Lower IC_50_ values indicate stronger DPPH radical scavenging activity.

**Table 3:** TPC, TFC, and antioxidant activities of fortified yoghurts.

Sample	TPC (mg GAE/g)	TFC (mg CE/g)	DPPH (mg TE/g)	FRAP (mg TE/g)	CUPRAC (mg TE/g)
Control yoghurt	0.118 ± 0.021 ^f^	nd	0.542 ± 0.023 ^h^	0.260 ± 0.003 ^h^	0.720 ± 0.032 ^i^
NADES-PRE-10	0.148 ± 0.017 ^f^	0.055 ± 0.008 ^de^	0.557 ± 0.010 ^h^	0.393 ± 0.060 ^fgh^	1.004 ± 0.030 ^ghi^
NADES-PRE-20	0.173 ± 0.015 ^ef^	0.088 ± 0.008 ^cde^	0.573 ± 0.003 ^h^	0.631 ± 0.082 ^ef^	1.400 ± 0.064 ^fg^
NADES-PRE-30	0.244 ± 0.095 ^def^	0.117 ± 0.012 ^c^	0.608 ± 0.086 ^gh^	0.856 ± 0.033 ^de^	1.511 ± 0.060 ^ef^
Water-PRE-10	0.208 ± 0.017 ^def^	0.036 ± 0.000 ^e^	0.752 ± 0.026 ^e^	0.317 ± 0.018 ^gh^	0.853 ± 0.029 ^hi^
Water-PRE-20	0.282 ± 0.062 ^cde^	0.045 ± 0.003 ^e^	1.006 ± 0.040 ^d^	0.391 ± 0.025 ^fgh^	1.081 ± 0.038 ^fghi^
Water-PRE-30	0.390 ± 0.018 ^c^	0.080 ± 0.013 ^cde^	1.194 ± 0.113 ^c^	0.543 ± 0.065 ^fgh^	1.405 ± 0.045 ^fg^
NADES-POS-10	0.136 ± 0.012 ^f^	0.053 ± 0.001 ^de^	0.612 ± 0.021 ^gh^	0.567 ± 0.032 ^efg^	1.246 ± 0.043 ^fgh^
NADES-POS-20	0.167 ± 0.014 ^ef^	0.082 ± 0.019 ^cde^	0.629 ± 0.020 ^fgh^	1.006 ± 0.078 ^cd^	2.128 ± 0.156 ^d^
NADES-POS-30	0.208 ± 0.038 ^def^	0.109 ± 0.010 ^cd^	0.682 ± 0.015 ^efg^	1.244 ± 0.128 ^c^	2.815 ± 0.090 ^c^
Water-POS-10	0.189 ± 0.033 ^def^	0.030 ± 0.001 ^e^	0.586 ± 0.019 ^gh^	0.353 ± 0.027 ^fgh^	0.718 ± 0.070 ^i^
Water-POS-20	0.243 ± 0.036 ^def^	0.037 ± 0.002 ^e^	0.720 ± 0.043 ^ef^	0.425 ± 0.016 ^fgh^	1.105 ± 0.129 ^fghi^
Water-POS-30	0.305 ± 0.016 ^cd^	0.061 ± 0.008 ^cde^	0.934 ± 0.033 ^d^	0.563 ± 0.039 ^efg^	1.842 ± 0.535 ^de^

Notes: Values are expressed as mean ± standard deviation (*n* = 3). Different superscript letters indicate significant differences within the same column (*p* < 0.05). “nd” indicates not detected.

**Table 4:** Physicochemical properties of control and fortified yoghurts.

Sample	pH	Brix°	Viscosity at 350 s^−1^ (Pa.s)	L*	a*	b*	Syneresis (%)
Control yoghurt	4.583 ± 0.016 ^c^	10.798 ± 2.342 ^fg^	0.235 ± 0.004 ^a^	77.968 ± 0.359 ^a^	−3.218 ± 0.042 ^f^	7.507 ± 0.108 ^f^	5.490 ± 0.116 ^k^
NADES-PRE-10	4.625 ± 0.015 ^bc^	20.790 ± 0.374 ^e^	0.222 ± 0.004 ^a^	75.247 ± 1.246 ^b^	−2.460 ± 0.206 ^d^	7.478 ± 0.343 ^f^	6.604 ± 0.320 ^j^
NADES-PRE-20	4.647 ± 0.014 ^ab^	23.240 ± 0.376 ^d^	0.194 ± 0.007 ^bc^	70.643 ± 1.294 ^e^	−2.125 ± 0.032 ^c^	7.185 ± 0.026 ^g^	9.183 ± 0.444 ^h^
NADES-PRE-30	4.687 ± 0.010 ^a^	27.170 ± 0.825 ^bc^	0.176 ± 0.014 ^d^	64.120 ± 1.099 ^f^	−1.782 ± 0.193 ^b^	8.163 ± 0.078 ^d^	10.445 ± 0.212 ^f^
Water-PRE-10	4.428 ± 0.020 ^e^	15.507 ± 0.383 ^hi^	0.204 ± 0.009 ^b^	75.778 ± 0.480 ^b^	−2.483 ± 0.110 ^d^	8.152 ± 0.027 ^d^	8.300 ± 0.132 ^i^
Water-PRE-20	4.363 ± 0.029 ^f^	13.983 ± 0.567 ^ij^	0.148 ± 0.006 ^e^	72.773 ± 1.168 ^d^	−1.727 ± 0.281 ^b^	8.772 ± 0.162 ^bc^	13.670 ± 0.479 ^d^
Water-PRE-30	4.268 ± 0.035 ^h^	12.338 ± 0.287 ^j^	0.113 ± 0.005 ^g^	70.385 ± 0.520 ^e^	−1.453 ± 0.125 ^a^	9.075 ± 0.021 ^a^	18.822 ± 0.858 ^b^
NADES-POS-10	4.518 ± 0.015 ^d^	20.443 ± 0.618 ^ef^	0.190 ± 0.006 ^c^	75.488 ± 0.155 ^b^	−3.292 ± 0.067 ^f^	7.513 ± 0.080 ^f^	7.360 ± 0.093 ^j^
NADES-POS-20	4.448 ± 0.034 ^e^	25.988 ± 0.430 ^c^	0.130 ± 0.003 ^f^	73.167 ± 0.271 ^d^	−3.080 ± 0.025 ^ef^	7.592 ± 0.101 ^f^	10.060 ± 0.303 ^fg^
NADES-POS-30	4.373 ± 0.020 ^f^	28.665 ± 0.563 ^b^	0.098 ± 0.008 ^h^	71.168 ± 0.158 ^e^	−2.915 ± 0.030 ^e^	7.853 ± 0.066 ^e^	11.948 ± 0.139 ^e^
Water-POS-10	4.527 ± 0.010 ^d^	16.990 ± 1.097 ^gh^	0.156 ± 0.002 ^e^	76.368 ± 0.387 ^b^	−2.632 ± 0.054 ^d^	8.065 ± 0.047 ^de^	9.543 ± 0.180 ^gh^
Water-POS-20	4.455 ± 0.019 ^e^	16.967 ± 1.890 ^gh^	0.091 ± 0.008 ^h^	75.148 ± 0.343 ^bc^	−1.878 ± 0.102 ^bc^	8.545 ± 0.023 ^c^	14.808 ± 0.306 ^c^
Water-POS-30	4.320 ± 0.024 ^g^	15.315 ± 1.084 ^hi^	0.067 ± 0.003 ^i^	73.772 ± 0.360 ^cd^	−1.288 ± 0.153 ^a^	8.878 ± 0.082 ^ab^	20.835 ± 0.671 ^a^

Notes: Values are expressed as mean ± standard deviation (*n* = 3). Different superscript letters indicate significant differences within the same column (*p* < 0.05).

**Table 5:** Three-way ANOVA results for fortified yoghurt properties.

Response	Extract Type	Stage	Concentration	Extract × Stage	Extract × Concentration	Stage × Concentration	Extract × Stage × Concentration
TPC	<0.001 (0.613)	0.005 (0.125)	<0.001 (0.643)	0.027 (0.079)	0.021 (0.121)	0.035 (0.106)	0.919 (0.003)
TFC	<0.001 (0.982)	<0.001 (0.728)	<0.001 (0.985)	<0.001 (0.254)	<0.001 (0.819)	<0.001 (0.388)	0.010 (0.143)
DPPH	<0.001 (0.898)	<0.001 (0.751)	<0.001 (0.824)	<0.001 (0.508)	<0.001 (0.715)	0.053 (0.093)	0.095 (0.075)
FRAP	<0.001 (0.913)	<0.001 (0.714)	<0.001 (0.898)	<0.001 (0.629)	<0.001 (0.656)	0.006 (0.156)	0.002 (0.183)
CUPRAC	<0.001 (0.731)	<0.001 (0.656)	<0.001 (0.856)	<0.001 (0.517)	0.006 (0.159)	<0.001 (0.534)	0.049 (0.096)
pH	<0.001 (0.938)	<0.001 (0.709)	<0.001 (0.841)	<0.001 (0.927)	<0.001 (0.684)	<0.001 (0.627)	<0.001 (0.398)
°Brix	<0.001 (0.973)	<0.001 (0.713)	<0.001 (0.614)	0.679 (0.003)	<0.001 (0.858)	<0.001 (0.335)	0.102 (0.073)
Viscosity	<0.001 (0.739)	<0.001 (0.705)	<0.001 (0.964)	<0.001 (0.922)	<0.001 (0.874)	<0.001 (0.591)	<0.001 (0.796)
L*	<0.001 (0.752)	<0.001 (0.793)	<0.001 (0.924)	0.002 (0.150)	<0.001 (0.556)	<0.001 (0.672)	<0.001 (0.296)
a*	<0.001 (0.884)	<0.001 (0.802)	<0.001 (0.886)	<0.001 (0.771)	<0.001 (0.548)	0.634 (0.015)	0.001 (0.217)
b*	<0.001 (0.947)	0.034 (0.073)	<0.001 (0.868)	<0.001 (0.185)	<0.001 (0.588)	<0.001 (0.287)	<0.001 (0.325)
Syneresis	<0.001 (0.979)	<0.001 (0.737)	<0.001 (0.986)	0.034 (0.072)	<0.001 (0.931)	0.002 (0.184)	0.846 (0.006)

Notes: Values are presented as *p*-values, with partial eta squared (partial η^2^) values shown in parentheses. Partial η^2^ was calculated to indicate the magnitude of each main and interaction effect. Confidence intervals were not reported because the limited number of independent samples would result in imprecise interval estimates. Statistical significance was set at *p* < 0.05, and *p*-values reported as 0.000 in the statistical output are presented as *p* < 0.001. Extract type refers to NADES and water extracts; stage refers to PRE and POS fortification; and concentration refers to extract additions of 10%, 20%, and 30% (*v*/*v*).

## Data Availability

All data generated and analysed in this study are included in this article.

## Supplementary Materials

The following supporting information can be downloaded at: https://www.mdpi.com/article/10.3390/antiox15091126/s1, Table S1: Tentatively annotated phenolic compounds detected in NADES-based SunGold kiwifruit extract using LC-MS/MS. Figure S1: Pearson correlation plots between TPC and TFC of raw NADES and water extracts. Significant correlations are indicated at *p* < 0.05. Figure S2: Pearson correlation plots between TPC, TFC, and antioxidant activities of raw NADES and water extracts. Significant correlations are indicated at *p* < 0.05. Figure S3: Correlations among DPPH, FRAP, and CUPRAC values.
