# Supplementary material for: NADES-Extracted SunGold Kiwifruit Polyphenols as Functional Ingredients in Antioxidant-Enriched Yoghurt
Source: Antioxidants (Basel). 2026 Sep 5;15(9):1126. doi: 10.3390/antiox15091126 (PMC13603193; doi:10.3390/antiox15091126)
Supplement: Supplementary file 1 [file antioxidants-15-01126-s001.zip › antioxidants-4482137-supplementary.pdf]

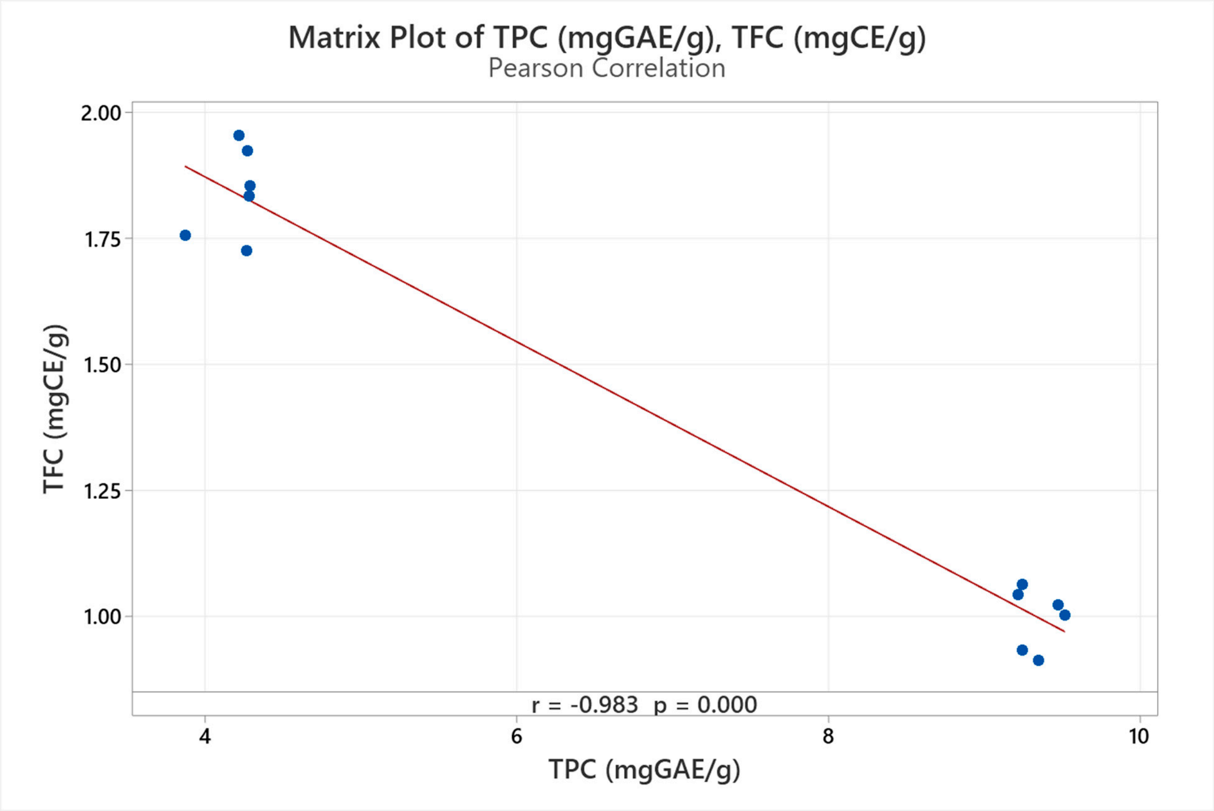

Figure S1. Pearson correlation plots between TPC and TFC of raw NADES and water extracts. Significant correlations are indicated at $p < 0.05$.

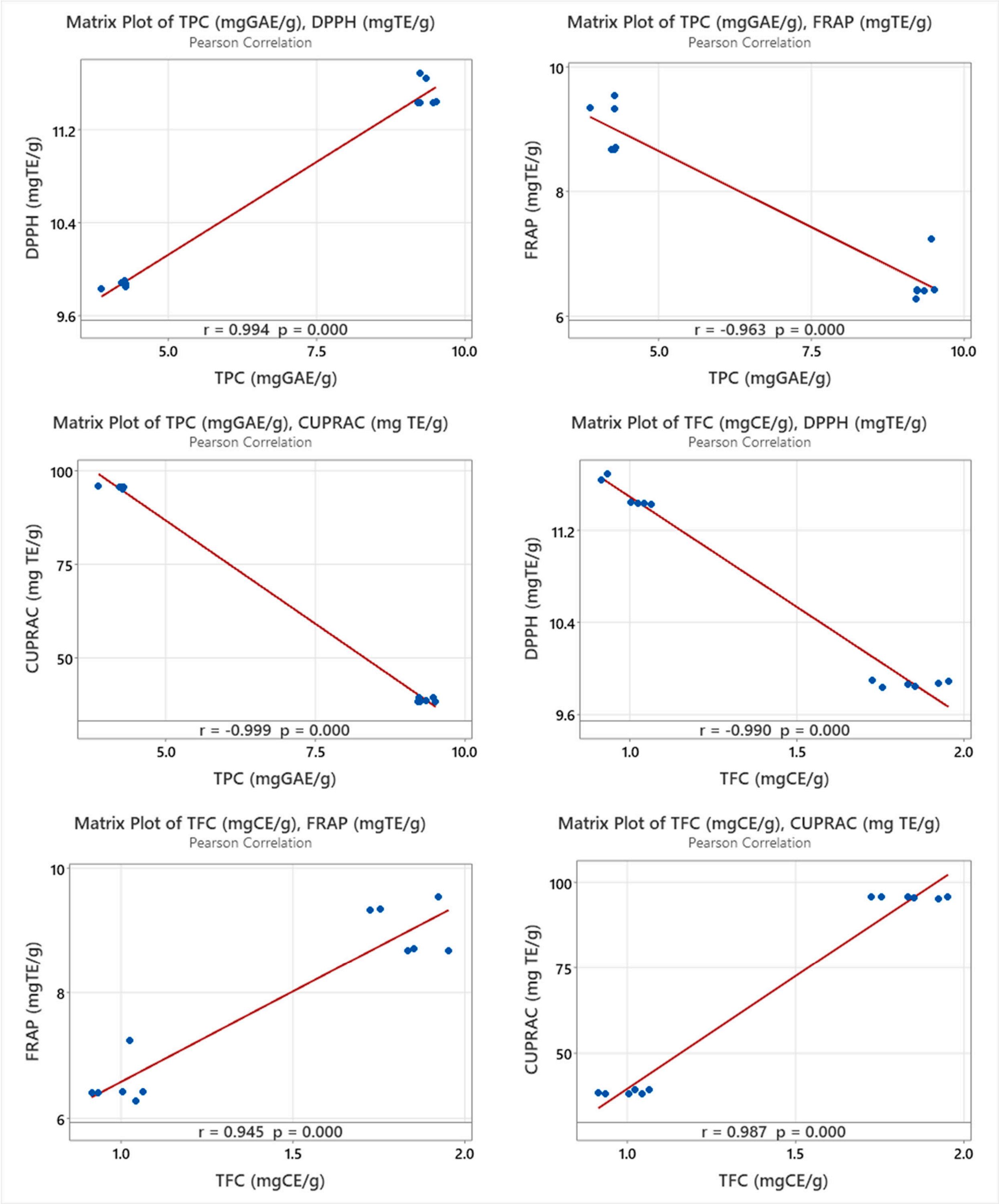

Figure S2. Pearson correlation plots between TPC, TFC, and antioxidant activities of raw NADES and water extracts. Significant correlations are indicated at $p < 0.05$.

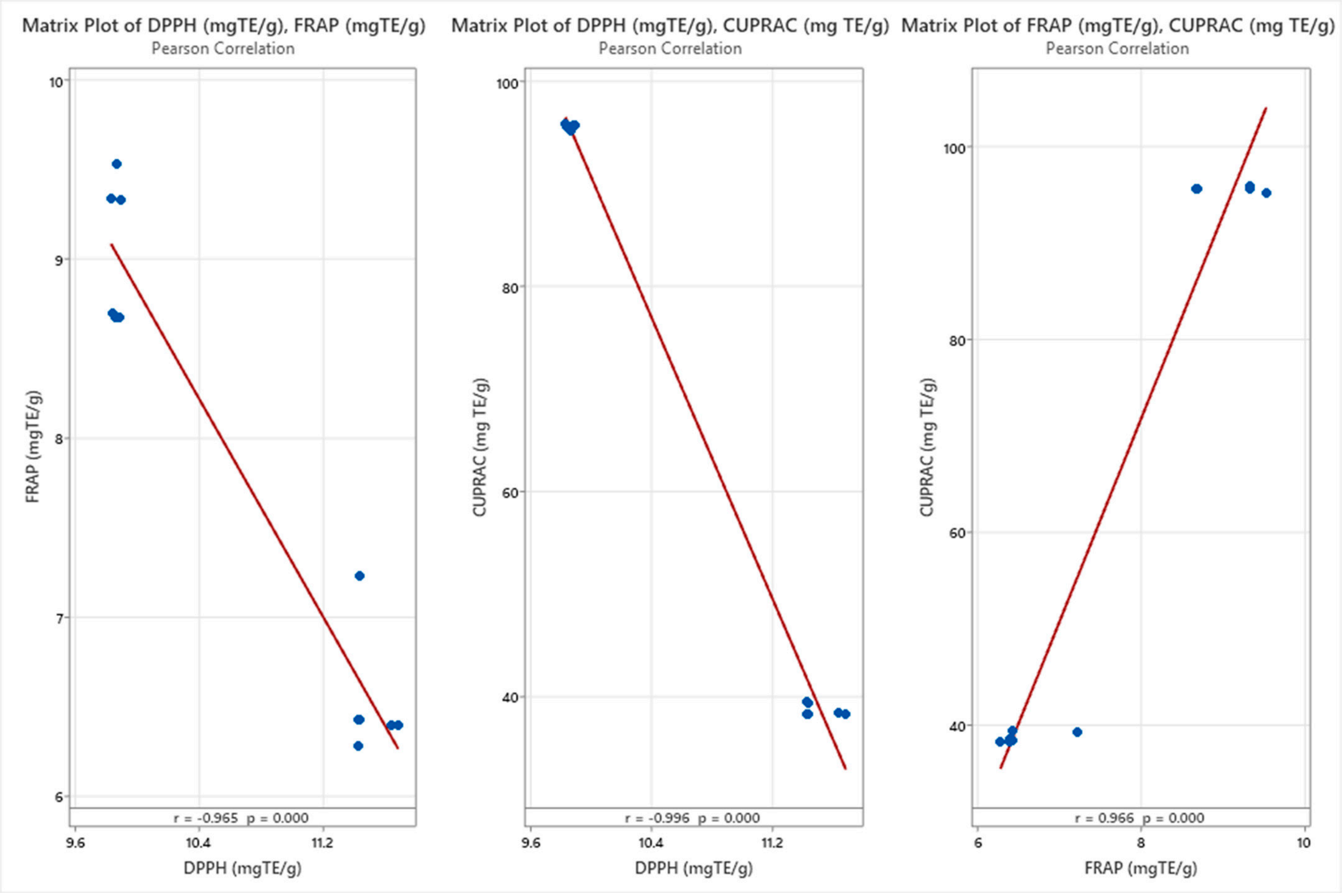

Figure S3. Correlations among antioxidant activities of raw SunGold kiwifruit extracts. Significant correlations are indicated at $p < 0.05$.

Table S1. Tentatively annotated phenolic compounds detected in NADES-based SunGold kiwifruit extract using LC-MS/MS.

Tentatively annotated compound	Class	Formula	RT (min)	m/z	Ion	Area
Catechin	Flavanol	$C_{15}H_{14}O_6$	13.223	289.07186	$[M–H]^-$	1,224,362,862
1-Caffeoyl-β-D-glucose	Hydroxycinnamic acid	$C_{15}H_{18}O_9$	11.821	341.08770	$[M–H]^-$	1,124,488,809
Caffeic acid	Hydroxycinnamic acid	$C_9H_8O_4$	10.515	179.03499	$[M–H]^-$	254,692,229
Chlorogenic acid	Hydroxycinnamic acid	$C_{16}H_{18}O_9$	5.439	353.08755	$[M–H]^-$	139,777,239
Procyanidin dimer B1	Proanthocyanidin	$C_{30}H_{26}O_{12}$	11.518	577.13568	$[M–H]^-$	83,703,187
4-Feruloylquinic acid	Hydroxycinnamic acid	$C_{17}H_{20}O_9$	10.781	367.10343	$[M–H]^-$	76,614,697
Epicatechin 7-O-glucuronide	Flavanol	$C_{21}H_{22}O_{12}$	6.843	465.10385	$[M–H]^-$	29,194,264
5-p-Coumaroylquinic acid	Hydroxycinnamic acid	$C_{16}H_{18}O_8$	8.992	337.09284	$[M–H]^-$	34,925,145
(+)-Gallocatechin	Flavanol	$C_{15}H_{14}O_7$	9.022	305.06668	$[M–H]^-$	23,799,313
Ferulic acid	Hydroxycinnamic acid	$C_{10}H_{10}O_4$	9.718	193.05071	$[M–H]^-$	7,654,476
Rutin	Flavonol glycoside	$C_{27}H_{30}O_{16}$	17.467	609.14631	$[M–H]^-$	7,394,420
Protocatechuic acid	Hydroxybenzoic acid	$C_7H_6O_4$	4.180	153.01941	$[M–H]^-$	388,084
Quercetin-3β-D-glucoside	Flavonol glycoside	$C_{21}H_{20}O_{12}$	17.663	463.08831	$[M–H]^-$	4,108,923
